# Maximum chest CT score is associated with progression to severe illness in patients with COVID-19: a retrospective study from Wuhan, China

**DOI:** 10.1186/s12879-020-05683-3

**Published:** 2020-12-11

**Authors:** Jianwei Xiao, Xiang Li, Yuanliang Xie, Zengfa Huang, Yi Ding, Shengchao Zhao, Pei Yang, Dan Du, Bin Liu, Xiang Wang

**Affiliations:** Department of Radiology, The Central Hospital of Wuhan, Tongji Medical College, Huazhong University of Science and Technology, 26 Shengli Avenue, Jiangan, Wuhan, 430014 Hubei China

**Keywords:** Chest CT, COVID-19, COPD, ROC, CT score, Multivariate regression

## Abstract

**Background:**

The Coronavirus Disease 2019 (COVID-19) pandemic is a world-wide health crisis. Limited information is available regarding which patients will experience more severe disease symptoms. We evaluated hospitalized patients who were initially diagnosed with moderate COVID-19 for clinical parameters and radiological feature that showed an association with progression to severe/critical symptoms.

**Methods:**

This study, a retrospective single-center study at the Central Hospital of Wuhan, enrolled 243 patients with confirmed COVID­19 pneumonia. Forty of these patients progressed from moderate to severe/critical symptoms during follow up. Demographic, clinical, laboratory, and radiological data were extracted from electronic medical records and compared between moderate- and severe/critical-type symptoms. Univariable and multivariable logistic regressions were used to identify the risk factors associated with symptom progression.

**Results:**

Patients with severe/critical symptoms were older (*p* < 0.001) and more often male (*p* = 0.046). A combination of chronic obstructive pulmonary disease (COPD) and high maximum chest computed tomography (CT) score was associated with disease progression. Maximum CT score (> 11) had the greatest predictive value for disease progression. The area under the receiver operating characteristic curve was 0.861 (*95% confidence interval:* 0.811–0.902).

**Conclusions:**

Maximum CT score and COPD were associated with patient deterioration. Maximum CT score (> 11) was associated with severe illness.

**Supplementary Information:**

The online version contains supplementary material available at 10.1186/s12879-020-05683-3.

## Background

Worldwide, the Coronavirus Disease 2019 (COVID-19) pandemic, caused by infection with the novel Severe Acute Respiratory Syndrome Coronavirus 2 (SARS-CoV2), has resulted (as of October 23, 2020) in 41,570,883 confirmed cases and 1,134,940 deaths [1]. As of that date, the rate of overall case-fatality across China was approximately 5.1%. Although relatively few deaths have been observed in patients with mild COVID-19, the rate of case fatality clearly is elevated among critically ill patients. Nevertheless, the nature of the factors influencing the prognosis of COVID-19 patients remains unclear [2, 3].

The onset of COVID-19 is associated with symptoms such as fever, cough, and myalgia. The case definition adopted in China and elsewhere includes further stratification of cases as mild, moderate, and severe/critical [4]. More than 80% of the laboratory-confirmed cases in China (including both non-pneumonia and pneumonia cases) were of the mild to moderate types [5]. A fifth of these cases progressed to a severe or critical stage, with the highest reported case fatality rate reaching 4.47% for patients in Wuhan diagnosed before March 2020 [6]. Most previous studies have focused only on the general epidemiological findings, clinical characteristics, and outcomes of patients with COVID-19 [3, 7, 8]. However, few studies (to our knowledge) have investigated clinical findings in patients who progress from moderate- to severe/critical-type symptoms.

In this study, we performed a comprehensive analysis of the clinical course and imaging findings of 243 hospitalized COVID-19 patients who initially presented with moderate-type symptoms. These patients were admitted to the isolation ward of the Central Hospital of Wuhan; this facility was one of the first hospitals in Wuhan to admit COVID-19 patients, and is located very close to a seafood market suspected as a source of the initial outbreak. We sought to identify risk factors associated with progression from moderate- to severe/critical-type symptoms in COVID-19 patients.

## Methods

### Study design and participants

This research consisted of a retrospective single-center study at the Central Hospital of Wuhan. The study was approved (Approval No. 2020421) by the Central Hospital of Wuhan Ethics Committee; the requirement for written informed consent was waived by the Ethics Commission of the designated hospital based on the exigencies associated with emerging infectious diseases.

This study consecutively enrolled all patients at the Central Hospital of Wuhan who were diagnosed with COVID-19, based on WHO interim guidelines, between December 25, 2019, to February 16, 2020 [9]. The study design protocol was finalized before the initiation of data collection and parts of the patients have been included in our recent report [10]. Based on the new coronavirus pneumonia diagnosis and treatment protocols (version 6) developed by the National Health Commission of the People’s Republic of China (http://www.nhc.gov.cn/), the clinical classification of COVID-19 was stratified as follows: moderate-type cases included individuals with fever, respiratory tract involvement, and other symptoms, as well as imaging findings of pneumonia; severe-type cases additionally met any of the following criteria: (1) respiratory distress with a respiratory rate ≥ 30 beats/min; (2) oxygen saturation ≤ 93% at rest; and (3) arterial blood oxygen partial pressure (PaO_2_)/oxygen concentration (FiO_2_) ≤ 300 mmHg (1 mmHg = 0.133 kPa); critical-type cases additionally exhibited any of the following conditions: (1) respiratory failure requiring mechanical ventilation; (2) shock; (3) intensive care unit (ICU) admission for combined organ failure.

### Inclusion and exclusion criteria

Inclusion criteria were as follows: (1) diagnosis of COVID-19 based on exposure history or based on clinically compatible symptoms; (2) classification on admission as moderate type, according to protocols (version 6) developed by the National Health Commission of the People’s Republic of China; and (3) recovery during the study period. Exclusion criteria were as follows: (1) patient was still hospitalized at the end of follow-up; (2) patient transferred to another medical institution; (3) diagnosis not confirmed by repeated tests for the presence of SARS-CoV-2 RNA; (4) fatality; (5) patient diagnosed with severe-type or critical-type COVID-19 on admission; (6) incomplete medical records; or (7) case records lacked a second chest computed tomography (CT) scan.

### Data collection

Epidemiological, clinical, laboratory, and radiological characteristics, as well as outcome data, were extracted from electronic medical records. The data were reviewed by two physicians, and a third physician adjudicated any differences in interpretation between the two primary researchers. Fever was defined as axillary temperature higher than 37.3 °C. Epidemiological, clinical, and laboratory data were defined as the results of the first consultation or examination in the electronic medical records. The initial chest CT was defined as the first chest CT examination at or following admission.

### CT acquisition and evaluation

Chest CT scans were performed with the patient in a supine position and using a single inspiratory phase on one of the four CT systems available at our facility. These machines included the following equipment: Bright Speed Elite (GE, America); Philips Ingenuity Core128 (Philips Medical Systems, Best, the Netherlands); uCT 760 (United Imaging, China); and SOMATOM Definition AS (Siemens Healthineers, Germany). The following primary settings were used: tube voltage, 120 kVp; automatic tube current modulation (20–130 mAs); matrix, 512 × 512; field of view (FOV), 350 mm × 350 mm - 370 mm × 370 mm; pitch, 0.75–1.25 mm; and slice thickness, 1–1.5 mm. The reconstructed images were sent automatically to the corresponding post-processing workstation and Picture Archiving and Communication Systems (PACS) for multiplanar reconstruction post-processing. All chest CT images were analyzed independently, and the features were scored by a senior thoracic radiologist with more than 20 years of experience who was blinded to clinical and laboratory findings. A previously described semi-quantitative scoring system was used to estimate the pulmonary involvement of lesions on the basis of the area involved [11]. Briefly, each of the 5 lung lobes was visually scored from 0 to 5, as follows: 0, no involvement; 1, < 5% area involvement; 2, 5–25% area involvement; 3, 26–49% area involvement; 4, 50–75% area involvement; and 5, > 75% area involvement. The total CT score was calculated as the sum of the scores for the 5 lung lobes in a given case, yielding a value that ranged from 0 to 25. All CT scans acquired after the development of severe disease were excluded.

### Endpoints

After admission, patients were subjected to re-examination by performing a second chest CT scan. Serial chest CT scans were performed based on the hospital’s clinical decision policy. The primary endpoint of the present study was the development of severe/critical illness. The secondary endpoint was recovery from COVID-19.

### Statistical analyses

Continuous variables are presented as mean ± SD or median with interquartile range (IQR), depending on the normality of distribution. Categorical variables are presented as frequencies and percentages. Continuous variables were compared between the moderate-type group and the severe/critical-type group using a non-paired Student’s t-test or Mann-Whitney U test. Categorical variables were compared using a chi-squared test or Fisher’s exact test, as appropriate. Univariable and multivariable logistic regression models were used to explore the risk factors associated with progression from moderate-type symptoms to severe/critical-type symptoms. Considering the total number of severe/critical-type cases (*n* = 40) in the present study, and to avoid overfitting in the model, we excluded variables if any of the following conditions applied: between-group differences in variables were not significant; variable accuracy was unconfirmed (e.g., surgery history, which was self-reported); number of events was too small to calculate an odds ratio; or variables exhibited collinearity with the maximum CT score. Using the new coronavirus pneumonia diagnosis protocols as the standard of reference, diagnostic performance with the highest CT score of cases that progressed to severe/critical type was determined using analyses of sensitivity, specificity, positive predictive value (PPV), negative predictive value (NPV), accuracy, and area under the curve (AUC). The 95% confidence intervals (CIs) also were reported. Diagnostic ability was illustrated using a receiver operating characteristic curve (ROC). A two-tailed *p* < 0.05 was considered statistically significant. All statistical analyses were performed using SPSS, version 18 (SPSS, Inc., Chicago, IL).

## Results

**Fig. 1:**
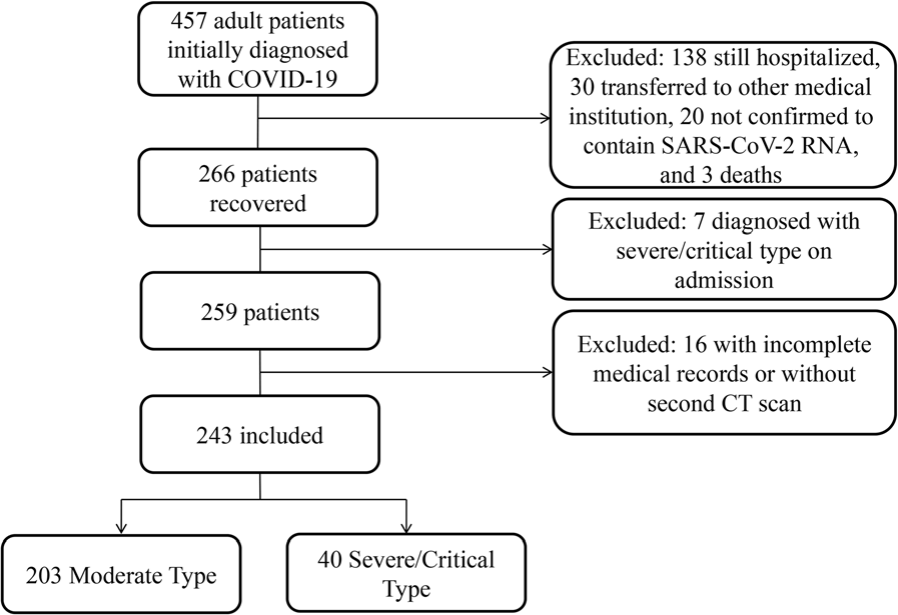
Flow chart showing inclusion of patients in the present study

A total of 457 adult patients diagnosed with COVID-19 were admitted to our hospital from December 25, 2019, to February 16, 2020. Of these, 191 patients remained hospitalized, had diagnoses that were not confirmed by SARS-CoV-2 RNA detection, or had died by the time of our final follow-up; these cases were excluded from our analysis. We also excluded another 7 patients who were diagnosed with severe/critical-type symptoms on admission. An additional 16 patients were excluded because their medical records were not available. In total, 243 patients with confirmed COVID­19 pneumonia were enrolled in the analysis (Fig. 1). Forty patients progressed from having moderate symptoms to severe/critical symptoms during follow-up (Supplementary file). The mean age of these 243 patients was 47 years, with a range of 20 to 89 years. One hundred thirty-eight (56.8%) were female (Table 1). The patients who developed severe/critical symptoms were older (*p* < 0.001) and more likely male (*p* = 0.046) than patients with moderate-type symptoms. The most common symptoms at onset were fever (83.5%) and cough (63.8%), followed by weakness (45.7%) and sputum (28.8%). Chest tightness was more likely to occur in patients with severe/critical symptoms (42.5%) than in patients with (*p* = 0.002). Patients in the severe/critical-type symptom group had (compared to patients in the moderate-type group) significantly higher mean serum levels of D-dimer (2.1 ± 4 vs. 0.8 ± 1.6 mg/L; *p* < 0.001), C-reactive protein (3.6 ± 3.2 vs. 2 ± 3 mg/L; *p* = 0.038), α-hydroxybutyrate dehydrogenase (247 ± 352 vs. 142 ± 57 U/L; *p* < 0.001), lactate dehydrogenase (251 ± 119 vs. 185 ± 79 U/L; *p* = 0.002), creatine kinase, (273 ± 636 vs. 120 ± 172 U/L; *p* < 0.001), and creatinine (211 ± 449 vs. 67 ± 43 μmol/L; *p* < 0.001). The neutrophil-to-lymphocyte ratio (NLR) also was significantly higher in the severe/critical-type group than in the moderate-type group (10.6 ± 7.7 vs 3.7 ± 3.9; *p* < 0.001). Other laboratory findings did not differ significantly between the two groups. In comparison to patients in the moderate-type group, patients in the severe/critical-type group were more likely to have underlying hypertension (15, 37.5% vs. 30, 14.8%, respectively), chronic obstructive pulmonary disease (COPD) (5, 12.5% vs. 5, 2.5%), and chronic kidney disease (6, 15% vs. 4, 2%).

**Table 1:** Clinical characteristics, laboratory findings, and imaging features of 243 COVID-19 patients

	Total (*n* = 243)	Moderate Type (*n* = 203)	Severe/Critical Type (n = 40)	*p* value
**Characteristics**				
Age, years	47.0 (20–89)	44.7 (20–87)	58.7 (28–89)	< 0.001
< 60	175 (72)	154 (75.9)	21 (52.5)	0.003
≥ 60	68 (28)	49 (24.1)	19 (47.5)	
Sex				0.046
Male	105 (43.2)	82 (40.4)	23 (57.5)	
Female	138 (56.8)	121 (59.6)	17 (42.5)	
**Symptoms**				
Fever	185 (83.5)	158 (77.8)	27 (67.5)	0.161
Cough	155 (63.8)	130 (64)	25 (62.5)	0.853
Sputum	70 (28.8)	56 (27.6)	14 (35)	0.344
Weakness	111 (45.7)	90 (44.3)	21 (54.5)	0.343
Diarrhea	21 (8.6)	18 (8.9)	3 (7.5)	0.779
Nausea/Vomiting	20 (8.2)	14 (6.9)	6 (15)	0.088
Chest tightness	57 (23.5)	40 (19.7)	17 (42.5)	0.002
Dyspnea	12 (4.9)	9 (4.4)	3 (7.5)	0.413
Myalgia	66 (27.2)	58 (28.6)	8 (20)	0.265
Chill	41 (16.9)	36 (17.7)	5 (12.5)	0.419
Conjunctival congestion	1 (0.4)	1 (0.5)	0	0.835
Dizziness	45 (18.5)	37 (18.2)	8 (20)	0.792
**Laboratory findings**				
White blood cell count, ×10^9^/L	5 (2.1)	4.8 (1.9)	5.9 (2.8)	0.006
< 10	236 (97.1)	199 (98)	37 (92.5)	0.056
≥ 10	7 (2.9	4 (2)	3 (7.5)	
Lymphocyte count, ×10^9^/L	1.2 (0.6)	1.2 (0.6)	0.9 (0.4)	0.07
< 1	110 (45.3)	87 (42.9)	23 (57.5)	0.089
≥ 1	133 (54.7)	116 (57.1)	17 (42.5)	
Monocyte count, ×10^9^/L	0.4 (0.2)	0.4 (0.2)	0.4 (0.3)	0.02
< 0.5	187 (77)	158 (77.8)	29 (72.5)	0.464
≥ 0.5	56 (23)	45 (22.2)	11 (27.5)	
Platelet, ×10^9^/L	182 (66)	183 (66)	172 (67)	0.414
< 100	19 (7.8)	15 (7.4)	4 (10)	0.574
≥ 100	224 (92.2)	188 (92.6)	36 (90)	
Hemoglobin, g/L	133 (26)	133 (28)	131 (19)	0.924
D-dimer, mg/L	1 (2.2)	0.8 (1.6)	2.1 (4)	< 0.001
< 1	199 (81.9)	173 (85.2)	26 (65)	0.002
≥ 1	44 (18.1)	30 (14.8)	14 (35)	
C-reactive protein, mg/L	2.3 (3.1)	2 (3)	3.6 (3.2)	0.038
< 1	121 (49.8)	107 (52.7)	14 (35)	0.041
≥ 1	122 (50.2)	96 (47.3)	26 (65)	
α-Hydroxybutyrate dehydrogenase, U/L	161 (161)	142 (57)	247 (352)	< 0.001
< 180	195 (80.2)	173 (85.2)	22 (55)	< 0.001
≥ 180	48 (19.8)	30 (14.8)	18 (45)	
Lactate dehydrogenase, U/L	197 (91)	185 (79)	251 (119)	0.002
< 250	199 (81.9)	178 (87.7)	21 (52.5)	< 0.001
≥ 250	44 (18.1)	25 (12.3)	19 (47.5)	
Creatine kinase, U/L	148 (313)	120 (172)	273 (636)	< 0.001
< 190	214 (88.1)	184 (90.6)	30 (75)	0.005
≥ 190	29 (11.9)	19 (9.4)	10 (25)	
Alanine aminotransferase, U/L	30 (45)	30 (48)	31 (26)	0.662
Aspartate aminotransferase, U/L	28 (23)	27 (22)	34 (25)	0.526
γ-Glutamyltransferase, U/L	37 (52)	34 (50)	54 (60)	0.07
Blood urea nitrogen, mmol/L	7.1 (28)	6.7 (30)	9.2 (11)	0.744
Creatinine, μmol/L	90.7 (192)	67 (43)	211 (449)	< 0.001
< 97	226 (93)	193 (95.1)	33 (82.5)	0.004
≥ 97	17 (7)	10 (4.9)	7 (17.5)	
Procalcitonin, ng/mL	0.1 (0.2)	0.09 (0.18)	0.16 (0.28)	0.135
Brain natriuretic peptide	420 (2587)	69 (109)	1532 (5210)	< 0.001
NLR	3.9 (4.2)	3.7 (3.9)	10.6 (7.7)	< 0.001
**Comorbidities**				
Any	97 (40.2)	70 (34.8)	27 (67.5)	< 0.001
Hypertension	45 (18.5)	30 (14.8)	15 (37.5)	0.001
Diabetes	24 (9.9)	18 (8.9)	6 (15)	0.235
Hyperlipemia	10 (4.1)	8 (3.9)	2 (5)	0.671
Chronic obstructive pulmonary disease	10 (4.1)	5 (2.5)	5 (12.5)	0.003
Chronic pulmonary disease	12 (4.9)	8 (3.9)	4 (10)	0.116
Cerebrovascular disease	1 (0.4)	1 (0.5)	0	0.036
Chronic kidney disease	10 (4.1)	4 (2)	6 (15)	0.002
Fatty liver	12 (5)	9 (4.5)	3 (7.5)	0.316
Hepatitis	6 (2.5)	3 (1.5)	3 (7.5)	0.058
Malignancy	9 (3.7)	8 (4)	1 (2.5)	0.656
Surgery history	34 (14)	27 (13.3)	7 (17.5)	0.484
**Imaging features**				
Initial CT score, median (IQR)	3 (0–15)	3 (0–15)	3 (1–15)	0.112
Maximum CT score, median (IQR)	8 (0–28)	7 (0–20)	14 (3–28)	< 0.001
**Others**				
Onset of symptoms to hospital, median (IQR), days	4 (2–7)	4 (2–7)	4 (2–7)	0.733
Hospital stay, median (IQR), days	19 (13–25)	18 (13–24)	22 (18.5–28.5)	< 0.001
Time from illness onset to highest CT score, median (IQR), days	10 (6–13)	9 (6–12)	13 (8–17.8)	< 0.001

Data are mean (SD), median (IQR), n (%), or n/N (%). *NLR* Neutrophil Lymphocyte Ratio; *SD* standard deviation; *OR* odds ratio; *IQR* interquartile range

**Fig. 2:**
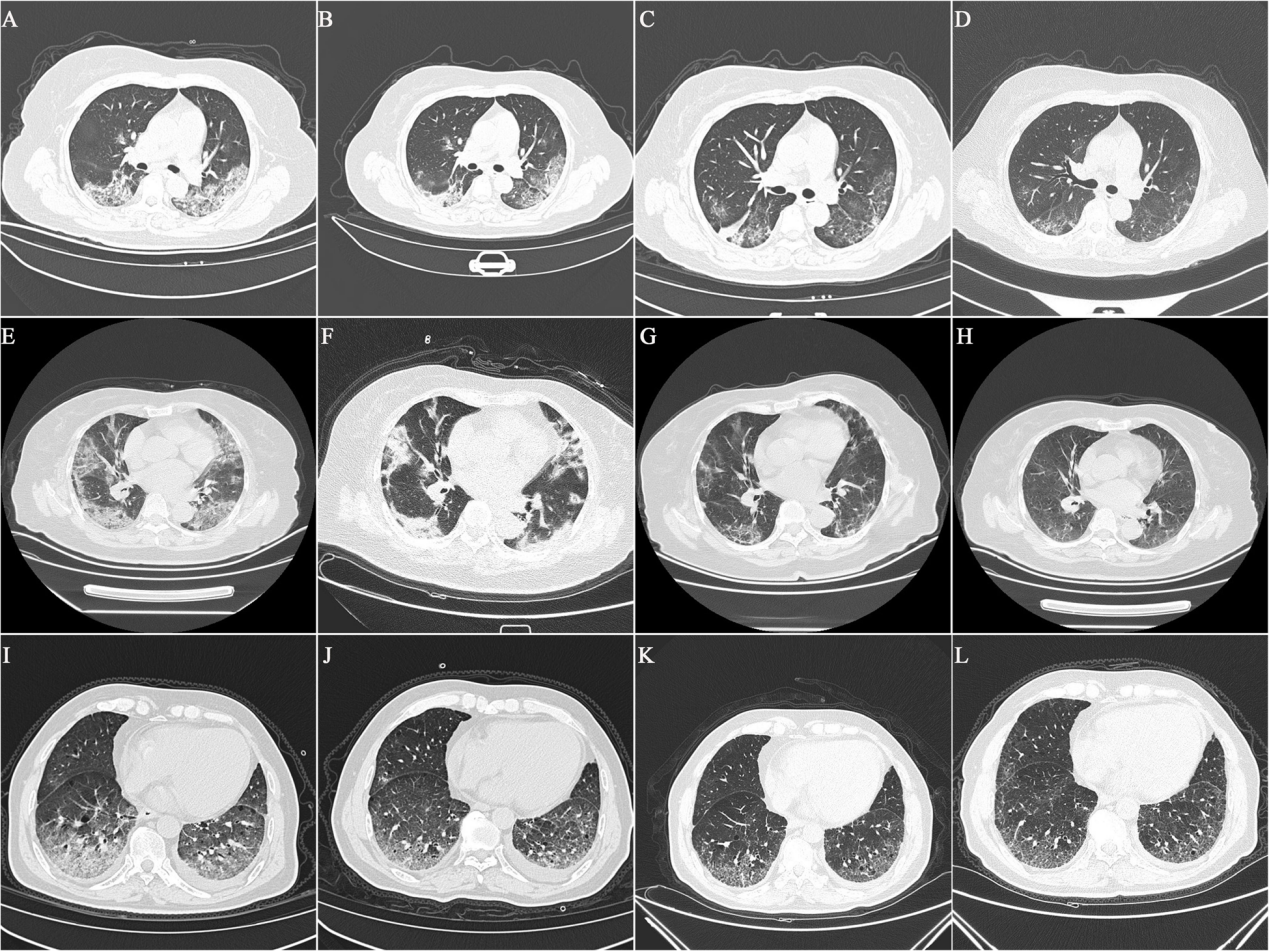
Chest CT images in moderate-type group (A-D), severe-type group (E-H), and critical-type group (I-L)

**Fig. 3:**
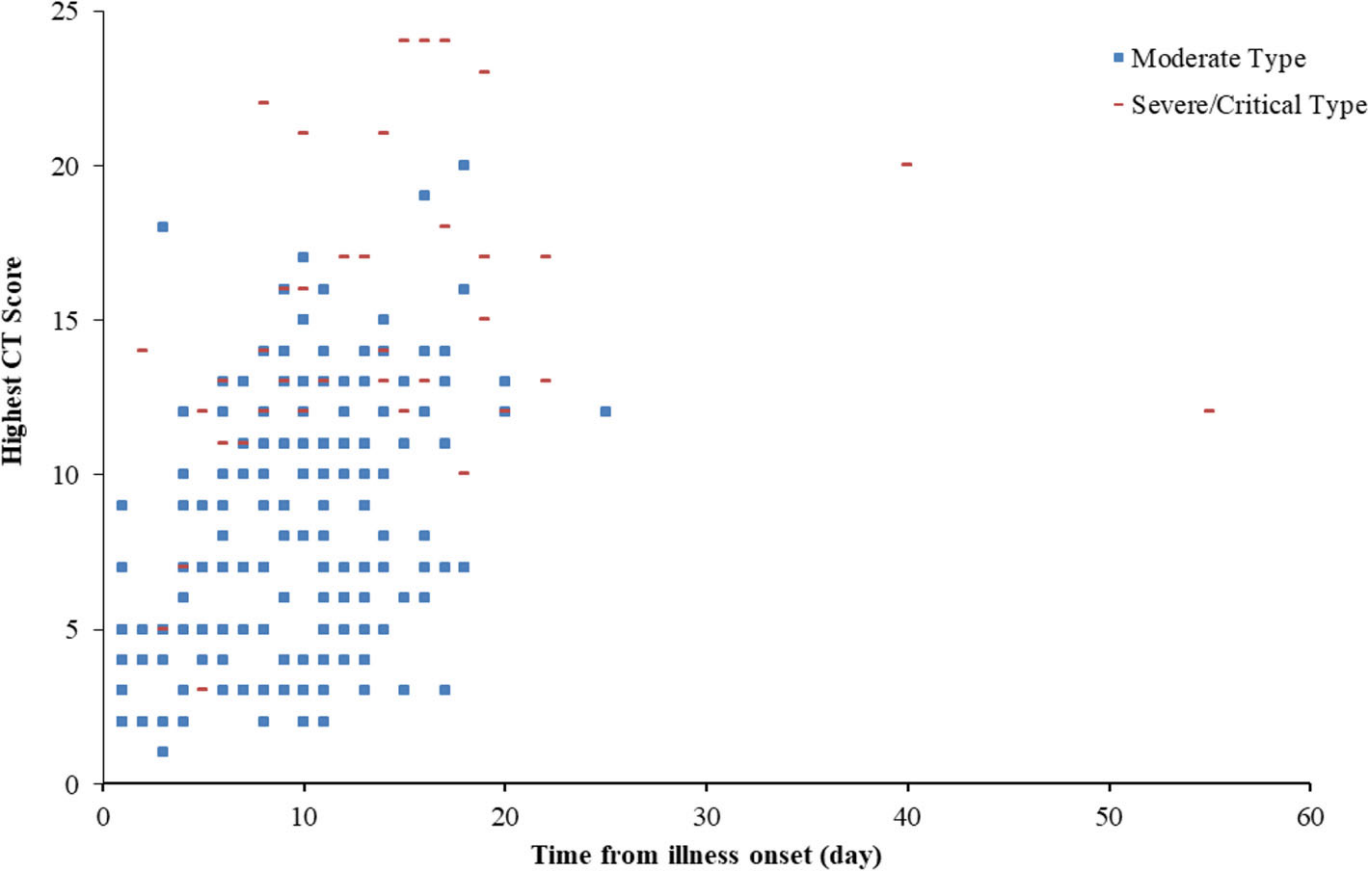
Scatter diagram of maximum CT score with time from illness onset in the two groups

All patients had abnormal CT imaging features (Fig. 2). There were no significant differences in the initial CT scores between the moderate-type group and severe/critical-type group (*p* = 0.112). Compared to the moderate-type group, patients in the severe/critical-type group had significantly higher median maximum CT score (7, IQR 0–20, vs. 14, IQR 3–28, respectively; *p* < 0.001). There was no significant difference in the median time from onset of symptoms to hospitalization between the moderate-type group and the severe/critical-type group (*p* = 0.733). In comparison with patients in the moderate-type group, patients in the severe/critical-type group had longer hospital stays (22 days, IQR 18.5–28.5, vs. 18 days, IQR 13–24, respectively; *p* < 0.001) and a longer time from illness onset to high CT score (13 days, IQR 8–17.8, vs. 9 days, IQR 6–12; *p* < 0.001) (Fig. 3).

Using univariable analysis, the calculated odds of progression to the severe/critical-type symptoms was found to be higher in patients having any comorbidity, including hypertension and COPD. Age (≥60 years); sex (male); chest tightness; serum levels of D-dimer (≥1 mg/L), C-reactive protein (≥1 mg/L), α-hydroxybutyrate dehydrogenase (≥180 U/L), lactate dehydrogenase (≥250 U/L), creatine kinase (≥190 U/L), and creatinine (≥97 μmol/L); serum NLR; and highest CT score also were associated with disease progression. The length of the hospital stay and time from illness onset to the time of the highest CT score also were associated with disease progression. No collinearity was detected between variables. Next, we included these variables in the multivariable logistic regression analysis. We found that the combination of COPD (*OR* = 9.06, *95% CI* [1.30–63.36]; *p* = 0.026) and a higher maximum CT score (*OR* = 1.39, *95% CI* [1.21–1.60]; *p* < 0.001) were associated with disease progression (Table 2).

**Table 2:** Risk factors associated with deterioration

	Univariable *OR (95% CI)*	*p* value	Multivariable *OR (95% CI)*	*p* value
**Characteristics**				
Age, years				
< 60	1 (ref)		1 (ref)	
≥ 60	2.84 (1.41–5.72)	0.003	1.48 (0.54–4.05)	0.444
Sex (vs. female)	2.00 (1.01–3.97)	0.048		
**Symptoms**	0			
Chest tightness	3.01 (1.47–6.16)	0.003	0.85 (0.32–2.78)	0.918
**Laboratory findings**				
White blood cell count, ×10^9^/L				
< 10	1 (ref)			
≥ 10	1.03 (0.87–18.77)	0.075		
Lymphocyte count, ×10^9^/L				
< 1	1 (ref)			
≥ 1	0.55 (0.28–1.10)	0.092		
Monocyte count, ×10^9^/L				
< 0.5	1 (ref)			
≥ 0.5	1.33 (0.62–2.87)	0.47		
D-dimer, mg/L				
< 1	1 (ref)		1 (ref)	
≥ 1	3.11 (1.46–6.62)	0.003	1.82 (0.62–0.21)	0.388
C-reactive protein, mg/L				
< 1	1 (ref)			
≥ 1	2.07 (1.02–4.19)	0.043	0.62 (0.21–1.84)	0.276
α-hydroxybutyrate dehydrogenase, U/L				
< 180	1 (ref)			
≥ 180	4.72 (2.27–9.83)	< 0.001		
Lactate dehydrogenase, U/L				
< 250	1 (ref)			
≥ 250	6.44 (3.05–13.62)	< 0.001		
Creatine kinase, U/L				
< 190	1 (ref)		1 (ref)	
≥ 190	3.23 (1.37–7.61)	0.007	1.20 (0.31–4.63)	0.792
Creatinine, μmol/L				
< 97	1 (ref)		1 (ref)	
≥ 97	4.09 (1.46–11.51)	0.008	2.43 (0.56–10.50)	0.235
NLR	1.13 (1.04–1.23)	0.003	1.09 (0.98–1.21)	0.105
**Comorbidities (vs no)**				
Any	3.89 (1.89–8.01)	< 0.001	1.95 (0.58–6.51)	0.278
Hypertension	3.46 (1.64–7.31)	0.001	0.67 (0.17–2.61)	0.567
Chronic obstructive pulmonary disease	5.66 (1.56–20.56)	0.008	9.06 (1.30–63.36)	0.026
**Imaging features**				
Initial CT score, median (IQR)	1.07 (0.97–1.17)	0.184		
Maximum CT score, median (IQR)	1.40 (1.26–1.56)	< 0.001	1.39 (1.21–1.60)	< 0.001
**Others**				
Onset of symptoms to hospital, median (IQR), days	1.03 (0.94–1.13)	0.507		
Hospital stay, median (IQR), days	1.07 (1.03–1.12)	0.002		
Time from illness onset to highest CT score, median (IQR), days	1.15 (1.07–1.23)	< 0.001		

Data are mean (SD), median (IQR), *n (%)* or n/N (%). *NLR* Neutrophil Lymphocyte Ratio; *SD* standard deviation; *OR* odds ratio; *IQR* interquartile range; *CI* confidence interval

**Fig. 4:**
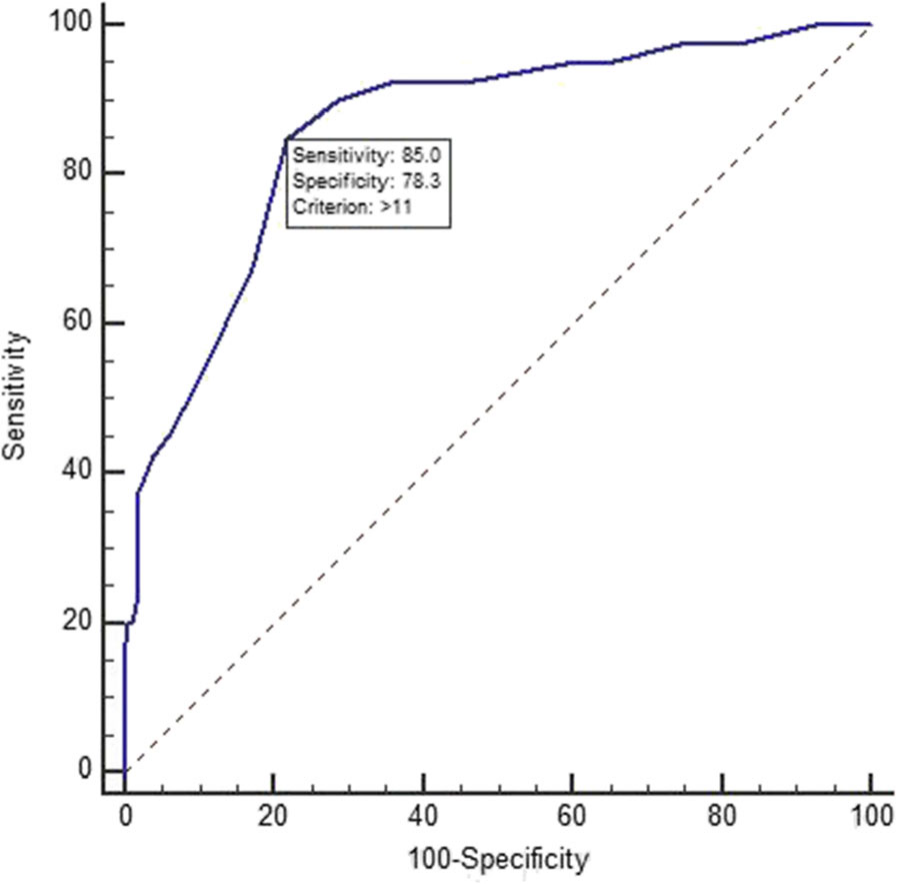
Receiver operating characteristic curve analyses of the highest CT score for prediction of disease deterioration

Using receiver operating characteristic curve (ROC) analysis, an optimal cutoff value of a maximum CT score of 11 with a sensitivity of 85.0% (*95% CI*: 70.2–94.3%) and specificity of 78.3% (*95% CI*: 72–83.8%) was shown to be predictive of disease progression (Fig. 4). The area under the ROC was 0.861 (*95% CI*: 0.811–0.902).

## Discussion

This retrospective study identified high CT scores and COPD as risk factors for deterioration in hospitalized patients with COVID-19 in Wuhan, China. Additionally, being older and male, having chest tightness, hypertension, and elevated serum levels of D-dimer, C-reactive protein, α-hydroxybutyrate dehydrogenase, lactate dehydrogenase, creatine kinase, creatinine, and serum NLR were associated with progression to severe or critical COVID-19 illness.

Recently, in a retrospective, multicenter cohort study of 191 patients, Zhou et al. demonstrated that older age is associated with mortality in hospitalized COVID-19 patients [12]. Other studies also have shown that older age (> 65 years) is associated with poorer clinical outcome in patients with COVID-19 [7, 13, 14]. The present study confirmed that more severely or critically ill patients were older (> 60 years) than patients with moderate-type COVID-19 symptoms. An age-dependent risk also has been seen in previous studies of severe acute respiratory syndrome (SARS) and middle east respiratory syndrome (MERS) [14, 15]. However, in the current study, older age was not shown to be an independent predictor of deterioration in hospitalized patients with COVID-19. The difference between the present and previous studies may be due, in part, to the different outcomes being assesssed. The current study was aimed at the progress of moderate-type patients with COVID-19 who eventually recovered, rather than dying. Furthermore, we had a relatively small sample size for the severe/critical group.

Chest CT is the routine imaging modality for clinical diagnosis of patients with COVID-19 pneumonia in the Hubei Province. This technique may help in screening patients with suspected COVID-19 symptoms, especially those with a negative reverse transcription-polymerase chain reaction (RT-PCR) result at the early stages of the disease [16]. In order to comprehensively evaluate the CT features of COVID-19 pneumonia, a semi-quantitative scoring system has been developed to quantitatively estimate the severity of inflammation based on quantifying the extent of pulmonary abnormalities (including ground-glass opacities, consolidations, or other fuzzy interstitial opacities) [11]. Using this method, no significant difference in initial CT score was found between moderate-type and severe/critical-type groups in the present study. In contrast, a recent study showed that CT scores in severe/critical-type groups were significantly higher than those in the moderate-type groups [17]. This difference may reflect the fact that all of the patients with COVID-19 in the present study were of the moderate type initially. With the progression of COVID-19, there were significant differences in the highest CT scores between the moderate-type and severe/critical-type groups. Moreover, using multivariable analysis, the maximum CT score were found to be an important independent predictor of deterioration of patients who progressed from moderate-type symptoms to severe/critical-type symptoms. Furthermore, ROC analysis showed that an optimal cutoff value of a maximum CT score index of 11 (sensitivity of 85.0% and specificity of 78.3%) predicted deterioration. We hope that this maximum CT score index can be used to identify patients at earlier stages of COVID-19 who potentially may progress to severe/critical-type symptoms from moderate-type symptoms. Patients with this maximum CT score index then would receive more aggressive treatment and close monitoring. However, the efficacy of such an approach remains to be validated in multi-center and large-sample studies in the future.

Lung complications, especially pneumonia, are common in patients with COPD. According to a national cross-sectional study, the total number of patients with COPD in China approximates 100 million [18]. Most recent studies have shown no significant differences in COVID-19 severity between patients with and without COPD [2, 8, 19–21]. In the current study, COPD was more common in the severe/critical-type group than in the moderate-type group, a finding that agrees with the results reported by Guan et al. [7]. In addition, COPD was found (in the present study) to be associated with the deterioration of patients with COVID-19. It should be noted that all studies to date, including the present work, have used small sample sizes of COVID-19 patients with COPD. The potential impact of COPD on the disease outcomes of patients with COVID-19 requires further observation and research.

Comorbidities have been shown to be associated with an increase in the risk of developing severe COVID-19. Recently published data suggested that neither type 2 diabetes mellitus nor hypertension are associated with the risk of developing severe COVID-19 [22]. A high incidence of COVID-19 was reported among colorectal cancer subjects and lung cancer patients [22]. Malignancy also was associated with a higher risk of severe COVID-19 [23]. Separately, COVID-19 patients with underlying liver disease were observed to have worse outcomes [24]. However, these comorbidities did not show statistical significance in our multivariable logistic regression analysis, presumably because of the relatively small sample size and few cases with complications.

Our study has some limitations. First, not all laboratory tests were done in all patients, including the measurement of levels of α-hydroxybutyrate dehydrogenase, lactate dehydrogenase, creatine kinase, procalcitonin and brain natriuretic peptide. Therefore, the roles of these parameters might be underestimated in predicting disease progression. In addition, we did not analyze the changes in laboratory findings in the process of the disease progression or patient recovery. Some of these results might also contribute to deterioration in some patients. Second, this was a retrospective study from a single center with a relatively small sample size and a certain selection bias, as some patients were transferred to other medical institutions by government decree. Thus, comparisons of clinical characteristics, laboratory findings, and imaging features may be skewed. In addition, chest CT score emerged as potentially associated with the risk for disease progression. However, there may have a risk of spurious association when multiple comparisons are made. Third, the semi-quantitative methods used for measuring COVID-19 pneumonia lesions may be somewhat subjective, and the time between the maximum chest CT score scan and the development of severe/critical illness was not analyzed as part of the present study. Last, infants, children and adolescents were not included in the present study; an effort should be made to include these groups in future studies.

## Conclusions

Maximum CT score and COPD showed independent association with an increased risk of COVID-19 disease progression. A maximum CT score higher than 11 was associated with development of severe illness.

## Supplementary Information

**Additional file 1.**

## Data Availability

The current manuscript describes the study protocol and all raw data is included within the supplementary file.

## Abbreviations

COVID­19: Coronavirus Disease 2019
CT: Computed Tomography
COPD: Chronic obstructive pulmonary disease
ROC: Receiver operating characteristic curve
NLR: Neutrophil Lymphocyte Ratio
IQR: Interquartile range
AUC: Area under the curve
CI: Confidence interval
PACS: Picture Archiving and Communication Systems
ICU: Intensive Care Unit

## References

[1] Weekly update on COVID-19 - 23 October 2020, https://www.who.int/publications/m/item/weekly-update-on-covid-19---23-october.

[2] Huang C, Wang Y, Li X, Ren L, Zhao J, Hu Y, Zhang L, Fan G, Xu J, Gu X (2020). Clinical features of patients infected with 2019 novel coronavirus in Wuhan, China. Lancet.

[3] Chen N, Zhou M, Dong X, Qu J, Gong F, Han Y, Qiu Y, Wang J, Liu Y, Wei Y (2020). Epidemiological and clinical characteristics of 99 cases of 2019 novel coronavirus pneumonia in Wuhan, China: a descriptive study. Lancet.

[4] Yang G, Wang Y, Zeng Y, Gao GF, Liang X, Zhou M, Wan X, Yu S, Jiang Y, Naghavi M (2013). Rapid health transition in China, 1990-2010: findings from the global burden of disease study 2010. Lancet.

[5] Diseases NCfC (2014). Report on cardiovascular diseases in China.

[6] Chen T, Wu D, Chen H, Yan W, Yang D, Chen G, Ma K, Xu D, Yu H, Wang H (2020). Clinical characteristics of 113 deceased patients with coronavirus disease 2019: retrospective study. BMJ.

[7] Guan WJ, Ni ZY, Hu Y, Liang WH, Ou CQ, He JX, Liu L, Shan H, Lei CL, Hui DSC, et al. Clinical characteristics of coronavirus disease 2019 in China. N Engl J Med. 2020;382(18):1708–20. 10.1056/NEJMoa2002032 PMC7092819 32109013

[8] Xu XW, Wu XX, Jiang XG, Xu KJ, Ying LJ, Ma CL, Li SB, Wang HY, Zhang S, Gao HN (2020). Clinical findings in a group of patients infected with the 2019 novel coronavirus (SARS-Cov-2) outside of Wuhan, China: retrospective case series. BMJ.

[9] DALYs GBD, Collaborators H (2016). Global, regional, and national disability-adjusted life-years (DALYs) for 315 diseases and injuries and healthy life expectancy (HALE), 1990-2015: a systematic analysis for the global burden of disease study 2015. Lancet.

[10] Wu G, Yang P, Xie Y, Woodruff HC, Rao X, Guiot J, Frix AN, Louis R, Moutschen M, Li J, et al. Development of a clinical decision support system for severity risk prediction and triage of COVID-19 patients at hospital admission: an international multicentre study. Eur Respir J. 2020;56(2):2001104. https://pubmed.ncbi.nlm.nih.gov/32616597/. https://erj.ersjournals.com/content/erj/56/2/2001104.full.pdf. 10.1183/13993003.01104-2020 PMC7331655 32616597

[11] Chang YC, Yu CJ, Chang SC, Galvin JR, Liu HM, Hsiao CH, Kuo PH, Chen KY, Franks TJ, Huang KM (2005). Pulmonary sequelae in convalescent patients after severe acute respiratory syndrome: evaluation with thin-section CT. Radiology.

[12] Zhou F, Yu T, Du R, Fan G, Liu Y, Liu Z, Xiang J, Wang Y, Song B, Gu X (2020). Clinical course and risk factors for mortality of adult inpatients with COVID-19 in Wuhan, China: a retrospective cohort study. Lancet.

[13] Wang D, Hu B, Hu C, Zhu F, Liu X, Zhang J, Wang B, Xiang H, Cheng Z, Xiong Y, et al. Clinical characteristics of 138 hospitalized patients with 2019 novel coronavirus-infected pneumonia in Wuhan, China. JAMA. 2020;323(11):1061–9. 10.1001/jama.2020.1585 PMC7042881 32031570

[14] Yang X, Yu Y, Xu J, Shu H, Xia J, Liu H, Wu Y, Zhang L, Yu Z, Fang M, et al. Clinical course and outcomes of critically ill patients with SARS-CoV-2 pneumonia in Wuhan, China: a single-centered, retrospective, observational study. Lancet Respir Med. 2020;8(5):475–81. 10.1016/S2213-2600(20)30079-5 PMC7102538 32105632

[15] Hong KH, Choi JP, Hong SH, Lee J, Kwon JS, Kim SM, Park SY, Rhee JY, Kim BN, Choi HJ (2018). Predictors of mortality in Middle East respiratory syndrome (MERS). Thorax.

[16] Xie X, Zhong Z, Zhao W, Zheng C, Wang F, Liu J. Chest CT for Typical Coronavirus Disease 2019 (COVID-19) Pneumonia: Relationship to Negative RT-PCR Testing. Radiology. 2020;296(2):E41-5. 10.1148/radiol.2020200343 PMC7233363 32049601

[17] Li K, Fang Y, Li W, Pan C, Qin P, Zhong Y, Liu X, Huang M, Liao Y, Li S. CT image visual quantitative evaluation and clinical classification of coronavirus disease (COVID-19). Eur Radiol. 2020;30(8):4407–16. 10.1007/s00330-020-06817-6 PMC7095246 32215691

[18] Wang C, Xu J, Yang L, Xu Y, Zhang X, Bai C, Kang J, Ran P, Shen H, Wen F (2018). Prevalence and risk factors of chronic obstructive pulmonary disease in China (the China pulmonary health [CPH] study): a national cross-sectional study. Lancet.

[19] Wang L, He W, Yu X, Hu D, Bao M, Liu H, Zhou J, Jiang H. Coronavirus disease 2019 in elderly patients: characteristics and prognostic factors based on 4-week follow-up. J Inf Secur. 2020;80(6):639–45. 10.1016/j.jinf.2020.03.019 PMC7118526 32240670

[20] Liu W, Tao ZW, Lei W, Ming-Li Y, Kui L, Ling Z, Shuang W, Yan D, Jing L, Liu HG, et al. Analysis of factors associated with disease outcomes in hospitalized patients with 2019 novel coronavirus disease. Chin Med J. 2020;133(9):1032–8. 10.1097/CM9.0000000000000775 PMC7147279 32118640

[21] Zhang JJ, Dong X, Cao YY, Yuan YD, Yang YB, Yan YQ, Akdis CA, Gao YD. Clinical characteristics of 140 patients infected with SARS-CoV-2 in Wuhan, China. Allergy. 2020;75(7):1730–41. 10.1111/all.14238 32077115

[22] Murillo-Zamora E, Trujillo X, Huerta M, Rios-Silva M, Mendoza-Cano O (2020). Male gender and kidney illness are associated with an increased risk of severe laboratory-confirmed coronavirus disease. BMC Infect Dis.

[23] Yarza R, Bover M, Paredes D, Lopez-Lopez F, Jara-Casas D, Castelo-Loureiro A, Baena J, Mazarico JM, Folgueira MD, Melendez-Carmona MA (2020). SARS-CoV-2 infection in cancer patients undergoing active treatment: analysis of clinical features and predictive factors for severe respiratory failure and death. Eur J Cancer.

[24] Ji D, Xu J, Qin E, Zhang D, Cheng G, Wang Y, Lau G (2020). Reply to: 'No evidence for an increased liver uptake of SARS-CoV-2 in metabolic-associated fatty liver disease'. J Hepatol.

